# In Vitro Antimicrobial Activity of *Embothrium coccineum* Used as Traditional Medicine in Patagonia against Multiresistant Bacteria

**DOI:** 10.3390/molecules21111441

**Published:** 2016-10-31

**Authors:** Nicole Canales, Iván Montenegro, Mario Párraga, Yusser Olguín, Patricio Godoy, Enrique Werner, Alejandro Madrid

**Affiliations:** 1 Escuela de Tecnología Médica Facultad de Ciencias de la Salud, Universidad Central de Chile, Edificio Gonzalo Hernández Uribe, Lord Cochrane 417, Santiago Centro 8320000, Chile; nicole.canales@alumnos.ucentral.cl; 2 Escuela de Obstetricia y Puericultura, Facultad de medicina, Campus de la Salud, Universidad de Valparaíso, Angamos 655, Reñaca, Viña del Mar 2520000, Chile; ivan.montenegro@uv.cl; 3 Centro de Investigaciones Biomédicas (CIB), Escuela de Medicina, Universidad de Valparaíso, Av. Hontaneda No. 2664, Valparaíso 2340000, Chile; mario.parraga@uv.cl; 4 Center for Integrative Medicine and Innovative Science (CIMIS), Facultad de Medicina, Universidad Andrés Bello, Santiago 8320000, Chile; yusser.olguin@unab.cl; 5 Instituto de Microbiología Clínica, Facultad de Medicina, Universidad Austral de Chile, Los Laureles s/n, Isla Teja, Valdivia 5090000, Chile; patricio.godoy@uach.cl; 6 Departamento de Ciencias Básicas, Campus Fernando May Universidad del Biobío, Avda. Andrés Bello s/n casilla 447, Chillán 3780000, Chile; ewerner@ubiobio.cl; 7 Departamento de Química, Facultad de Ciencias Naturales y Exactas, Universidad de Playa Ancha, Avda. Leopoldo Carvallo 270, Playa Ancha, Valparaíso 2340000, Chile; alejandro.madrid@upla.cl

**Keywords:** antimicrobial activity, sequential extracts, leaves, *Embothrium coccineum*, multiresistant bacteria

## Abstract

*Embothrium coccineum* J.R. Forst. & G. Forst is an evergreen tree that has been used as a folk remedy for the treatment of neuralgia, tooth pains, wound healing, and glandular conditions, as well as an antiseptic agent against bacterial infection. The antibacterial activities of sequential extracts (hexane, dichloromethane, ethyl acetate, and ethanol) from the leaves of *E. coccineum* were evaluated by means of the micro-dilution assay against six (*Escherichia coli*; *Klebsiella pneumoniae*; *Proteus mirabilis*; *Pseudomonas aeruginosa*; *Staphylococcus aureus* and *Streptococcus pyogenes*) multiresistant bacteria strains. Ethyl acetate extract showed the best spectra of antibacterial activity against all tested bacteria, and was analyzed by gas chromatography–mass spectrometry (GC-MS) for its composition. The results of the present work provide useful baseline information for the potential development and use of nanoparticles and/or nanofibers doped with extracts of *E. coccineum* in the fight against multiresistant bacteria, which would allow the validation of the traditional use of *E. coccineum* by native peoples of Patagonia as an antimicrobial agent in the biomedical Field.

## 1. Introduction

*Embothrium coccineum* J.R. Forst. & G. Forst belongs to the *Embothrium* genus in the family Proteaceae, which is an evergreen tree mainly distributed in the southern temperate regions of Argentina and Chile [1,2]. The aerial parts of *E. coccineum* have been used as a folk remedy for the treatment of neuralgia, tooth pains, wound healing, and glandular conditions, as well as an antiseptic agent in various cultures, for example among the Aónikenk, Huilliche, Kawésqar and Yagan people [3,4]. An earlier study by Mølgaard suggested that the ethanol extract of *E. coccineum* does not exhibit significant antibacterial activity against *E. coli* and *P. aeruginosa* [5], while in other studies it has been shown that extracts of *E. coccineum* have antioxidant activity because of their contents of phenols, flavonoids and anthraquinones, and molecules which were related to antimicrobial activity [6]. However, the available information concerning *E. coccineum* is particularly limited. Therefore, in view of the fact that *E. coccineum* has been widely used as a dermatologic drug [7], and therefore is likely safe and effective, and the importance of finding new anti-infective extracts to be loaded into or coated onto biomaterials, the aims of this study were to evaluate the antibacterial activities of sequential extracts from the leaves of *E. coccineum* against Gram-positive and Gram-negative multidrug-resistant strains and determine the composition of the most active extract by GC-MS.

## 2. Results

### 2.1. Extract Yields

The sequential method adapted to obtain different *E. coccineum* extracts was as follows: hexane (Hex), dichloromethane (DCM), ethyl acetate (AcOEt) and ethanol (EtOH). The highest yields were achieved with ethanol 12.62% (*w*/*w*), followed by hexane 5.49% (*w*/*w*), and dichloromethane 4.76% (*w*/*w*), while the lowest was ethyl acetate 3.55% (*w*/*w*).

### 2.2. Chemical Composition of AcOEt Extract

Results of the gas chromatographic analysis of ethyl acetate extract from the leaves of *E. coccineum* are summarized in Table 1.

Twenty-six components were identified in the ethyl acetate extract: 31.12% were hydroxybenzoic acids, 13.10% were terpenes, 13.06% were fatty acids and derivatives, 5.86% were aldehydes and ketones, 5.41% were linear hydrocarbons, 2.98% were lactones, 2.88% were aromatic hydrocarbons and 25.59% were unknown compounds. The ethyl acetate extract was mainly characterized by gentisic acid (21.17%), β-resorcylic acid (8.06%), phytol (7.19%), myristic acid (4.72%), and ethyl palmitate (3.89%). In addition, this is the first report made on the composition of leaves from *E. coccineum* in which hydroxybenzoic acids were the predominant portion.

### 2.3. In Vitro Antimicrobial Assay

A previous study showed that the chemical components of *E. coccineum* include anthraquinones, flavonoids and phenols [6]. It is known that secondary metabolites such as those described in *E. coccineum* have good antimicrobial properties. As shown in Table 2 and Table 3, the sequential extracts of the leaves showed antibacterial activity against all bacteria with minimum inhibitory concentration (MIC) values of 31.125–500 µg/mL and minimum bactericidal concentration (MBC) values of 62.5–500 µg/mL.

## 3. Discussion

The results revealed that the ethyl acetate extract showed higher activity against *S. pyogenes* with MIC and MBC values of 31.125–62.5 µg/mL; these values are comparable with those reported [8]. In addition, ethyl acetate extract was potently active against Gram-negative bacteria *K. pneumonia* with MIC and MBC values of 125 µg/mL in the clinical setting; it is the most significant member of the *Klebsiella* genus of Enterobacteriaceae [9]. The dichloromethane extract showed potent activity against *E. coli* with MIC and MBC values ranging from 31.125–62.5 µg/mL. Ethanol extract showed the same antibacterial activity against all bacteria with MIC and MBC values of 250–500 µg/mL. Similar results have been reported where the ethanol extract had low antimicrobial activity [10]. However, this study showed that the MIC and MBC values of *E. coccineum* extracts found highest against *S. aureus* were 250–500 µg/mL. Moreover, due to the activity shown by the ethyl acetate, it was subjected to GC-MS to identify the components in the extract responsible for the antibacterial activities observed in the study. Gentisic acid and β-resorcylic acid were the main metabolites in the extract; these hydroxybenzoic acids are found in many plants and fruits, and these compounds show a wide spectrum of action involving antitumor, antiviral, antibacterial, cardioprotective, pro-oxidant and antimutagenic activity [11]. The presence of the hydroxyl group and a system of delocalized electrons might be responsible for the antimicrobial activity of these compounds [12]. Furthermore, there is phytol in the extract, which is an important member of branched-chain unsaturated terpene and is a product of the chlorophyll metabolism in plants. It has also been reported that phytol can inhibit microorganisms [13]. In addition, the presence in the extract of fatty acids as myristic acid and ethyl palmitate, which are in natural fats and dietary oils, and are known to have antibacterial and antifungal properties [14], justifies the potential antimicrobial activity of this extract.

The results of the present work provide useful baseline information for the potential development and use of nanoparticles and/or nanofibers doped with extracts of *E. coccineum* in the fight against multiresistant bacteria, which would allow the validation of the traditional use of *E. coccineum* by native peoples of Patagonia as antimicrobial agents in the biomedical field.

## 4. Materials and Methods

### 4.1. Chemicals and Materials

Chemical reagents, culture media, solvents and positive controls were purchased from Sigma-Aldrich (Darmstadt, Germany).

### 4.2. Plant Material

*E. coccineum* was collected in November 2015 at Valdivia, Los Ríos Region, Chile. A voucher specimen (VALPL 2156) was deposited at the VALP Herbarium, Department of Biology, Universidad de Playa Ancha, Valparaíso, Chile.

### 4.3. Extraction

In brief, leaves of *E. coccineum* (500 g) were isolated manually from aerial parts and dried at room temperature for four weeks. The dry plant was powdered, and then mixed with 1.0 L hexane. The extraction was carried out by using an orbital shaker (150 rpm) at 25 °C for 72 h. The resulting mixture was filtered through Whatman No. 1 filter paper (Sigma-Aldrich, Darmstadt, Germany) and the hexane was removed from the filtrate under reduced pressure with a rotatory evaporator. The residue was further extracted with dichloromethane, ethyl acetate, and ethanol, sequentially and serially. Finally, each extract was weighed and the yield was calculated. *E. coccineum* extracts were kept at −4 °C prior to further analyses.

### 4.4. Chromatographic Analysis

The ethyl acetate extract was diluted with ethyl acetate, and analysis by gas chromatography (Hewlett Packard, Palo Alto, CA, USA) was carried out according to the method detailed elsewhere [15]. The operating conditions were as follows: on-column injection; injector temperature, 250 °C; detector temperature, 280 °C; carrier gas, He at 1.0 mL/min; oven temperature program: 40 °C increase to 260 °C at 4 °C /min, and then 260 °C for 5 min, to afford the best separation through a capillary Rtx-5MS column. The mass detector ionization employed an electron impact of 70 eV. Compounds in the chromatograms were identified by comparison of their mass spectra with those in the NIST/EPA/NIH Mass spectral Library [16]. Chromatographic peaks were considered “unknown”, when their similarity index (MATCH) and reverse similarity index (RMATCH) were less than 850 and discarded in this identification process [17]. These parameters are referred to the degree the target spectrum matches the standard spectrum in the NIST Library (the value 1000 indicates a perfect fit), and by comparison of their retention index with those reported in the literature [18], for the same type of column or those of commercial standards, when available. The retention indices were determined under the same operating conditions in relation to a homologous *n*-alkanes series (C_8_–C_36_) by the equation:

RI = 100 × (n + Tr_(unknown)_ − Tr_(n)_/Tr_(N)_ − Tr_(n)_) (1)

where, n = the number of carbon atoms in the smaller *n*-alkane; N = the number of carbon atoms in the larger *n*-alkane; and Tr = the retention time. Components relative concentrations were obtained by peak area normalization.

### 4.5. Bacterial Strains

The bacteria were recent clinical isolates, belonging to the Facultad de Ciencias de la Salud (Universidad Central de Chile) collection. They comprised of: *Escherichia coli, Klebsiella pneumoniae, Proteus mirabilis, Pseudomonas aeruginosa, Staphylococcus aureus, Streptococcus pyogenes.* All isolates were identified according to the latest Clinical and Laboratory Standards Institute (CLSI) guidelines [19]. Strains were cultured in Luria-Bertani (LB) broth, Mueller-Hinton Broth (MHB) and Mueller-Hinton agar, at 37 °C.

### 4.6. In Vitro Antimicrobial Assay

The Minimum Inhibitory Concentration (MIC) and minimum bactericidal concentration (MBC) of plant extract was determined using the broth dilution method, following the CLSI guidelines [20]. Briefly, the test extracts were first dissolved in dimethyl sulfoxide (DMSO) and the final concentration of DMSO in each medium was 1%, which did not affect the growth of the test strain. A dilution range of the extracts from 1 mg/mL to 12.5 µg/mL was tested. Later, 100 µL of microbial suspension (1 × 10^5^ CFU/mL) of each strain was inoculated in tubes with equal volume of nutrient broth and plant extracts. After, the tubes were incubated aerobically at 37 °C for 24 h. After the cultures were incubated at 37 °C for 24 h, the minimum inhibitory concentration (MIC) was determined as the lowest concentration of the test extract that demonstrated no visible growth. MBC value was determined by sub culturing the test dilution (100 μL) (which showed no visible turbidity) on to freshly prepared nutrient agar media. The plates were incubated further for 48 h at 37 °C. The highest dilution that yielded no single bacterial colony on the nutrient agar plates was taken as MBC. Rifampicin (1 mg/mL) and chloramphenicol (250 mg/mL) were used as positive control for bacterial strains and 1% DMSO was used as negative control. Each concentration of the extracts were tested in triplicate.

### 4.7. Statistical Analysis

Data were expressed as mean and standard deviation. A one-way variance was used to analyze data, with *p* < 0.05 representing a significant difference between means (Student’s *t*-test).

## 5. Conclusions

In conclusion, the obtained data indicate that the ethyl acetate extract showed the best spectra of antibacterial activity against all tested multiresistant bacteria. The biological activity could be partly explained by the majority presence of hydroxybenzoic acids, such as gentisic acid and β-resorcylic acid. Importantly, these results also contribute to the validation of *E. coccineum* as a medicinal plant for the treatment of infectious diseases.

## Figures and Tables

**Table 1:** Main components of AcOEt extract.

No.	RT (min)	Main Components	RI ^a^	% Area ^b^	Match	RIref ^c^
1	8.56	2-Benzylnaphthalene	350	0.88	865	349
2	8.74	Benzaldehyde	965	0.43	910	961
3	10.07	1,3-Dimethyladamantane	1130	1.10	955	1130
4	10.28	Benzoic Acid	1143	1.89	976	1143
5	10.66	2,6-Di-tert-butylbenzoquinone	1472	1.68	878	1468
6	10.90	Dihydroactinolide	1480	0.84	912	1479
7	11.48	Isolongifolan-8-ol	1515	4.81	970	1515
8	12.84	β-Resorcylic acid	1532	8.06	915	1532
9	13.99	9-Methyl-9H-fluorene	1593	0.78	890	1600
10	14.54	Hexadecane	1600	0.65	960	1600
11	14.63	Benzophenone	1625	2.72	905	1628
12	14.87	2-Pentadecanone	1680	1.03	945	1681
13	17.23	Heptadecane	1700	2.10	984	1700
14	17.66	1-Methylfluorene	1706	1.22	868	1709
15	18.92	Myristic acid	1768	4.72	943	1772
16	22.64	Gentisic acid	1794	21.17	905	1768
17	23.06	Octadecane	1800	0.83	980	1800
18	23.48	Phytol	1949	7.19	859	2076
19	24.33	Ethyl palmitate	1978	3.89	956	1980
20	24.52	Palmitic acid	1984	1.61	934	1983
21	24.72	Eicosane	2000	0.71	940	2000
22	25.92	Heptadecanoic acid	2022	0.94	876	2038
23	26.09	Ethyl linolenate	2198	1.90	961	2198
24	26.19	2-Coumaranone	2352	2.14	899	2352
25	26.45	Heptacosane	2700	0.69	989	2700
26	26.90	Octacosane	2800	0.43	985	2800

^a^ RI: Retention indices relative to C_8_–C_36_ *n*-alkanes on the Rtx-5MS capillary column; ^b^ Surface area of GC peak; ^c^ RIref: Retention indices reported in literature.

**Table 2:** Minimum inhibitory concentration of extracts from *E. coccineum*.

Sample	Antibacterial Test ^a^ (MIC μg/mL) ^#^ at 24 h					
	1	2	3	4	5	6
**Hex extract**	250 ± 0.0	250 ± 0.18	250 ± 0.10	250 ± 0.24	250 ± 0.05	62.5 ± 0.0
**DCM extract**	31.125 ± 0.2	250 ± 0.22	250 ± 0.11	250 ± 0.27	500 ± 0.07	62.5 ± 0.05
**AcOEt extract**	125 ± 0.40	125 ± 0.21	250 ± 0.15	250 ± 0.21	250 ± 0.04	31.125 ± 0.0
**ETOH extract**	250 ± 0.3	250 ± 0.15	250 ± 0.23	250 ± 0.09	250 ± 0.18	125 ± 0.07
**Rifampicin**	1.5 ± 0.05	1.0 ± 0.02	4.0 ± 0.32	31.125 ± 0.02	0.03 ± 0.01	0.06 ± 0.01
**Chloramphenicol**	0.25 ± 0.07	6.25 ± 0.13	R	2.0 ± 0.19	2.0 ± 0.08	4.0 ± 0.02
**DMSO**	i	i	i	i	i	i

i, inactive; R = Resistant; ^a^ **1**, *Escherichia coli*; **2**, *Klebsiella pneumoniae*; **3**, *Proteus mirabilis*; **4**, *Pseudomonas aeruginosa*; **5**, *Staphylococcus aureus*; **6**, *Streptococcus pyogenes*; **^#^** Mean of triplicates ± standard deviation of three replicates.

**Table 3:** Minimum bactericidal concentration of extracts from *E.* *coccineum*.

Sample	Antibacterial Test ^a^ (MBC μg/mL) ^#^ at 48 h					
	1	2	3	4	5	6
**Hex extract**	250 ± 0.45	250 ± 0.0	250 ± 0.30	250 ± 0.25	250 ± 0.13	62.5 ± 0.20
**DCM extract**	62.5 ± 0.16	250 ± 0.2	250 ± 0.40	250 ± 0.16	500 ± 0.32	125 ± 0.15
**AcOEt extract**	125 ± 0.23	125 ± 0.24	250 ± 0.25	250 ± 0.30	250 ± 030	62.5 ± 0.43
**ETOH extract**	500 ± 0.08	500 ± 0.12	250 ± 0.09	250 ± 0.28	250 ± 0.18	125 ± 0.34
**Rifampicin**	1.5 ± 0.02	2.0 ± 0.10	4.0 ± 0.19	62.5 ± 0.20	0.06 ± 0.0	0.12 ± 0.10
**Chloramphenicol**	0.25 ± 0.00	6.25 ± 0.0	R	2.0 ± 0.04	2.0 ± 0.10	4.0 ± 0.15
**DMSO**	i	i	i	i	i	i

i, inactive; R = Resistant; ^a^ **1**, *Escherichia coli* (1022); **2**, *Klebsiella pneumoniae* (1023); **3**, *Proteus mirabilis* (1024); **4**, *Pseudomonas aeruginosa* (1025); **5**, *Staphylococcus aureus* (1026); **6**, *Streptococcus pyogenes* (1027); **^#^** Mean of triplicates ± standard deviation of three replicates.
